# The Role and Mechanism of Berberine as a Candidate Drug for the Treatment of Radiation‐Induced Intestinal Injury

**DOI:** 10.1111/jcmm.71281

**Published:** 2026-08-09

**Authors:** Xihao Liu, Li Guo, Lu Guo, Lei Zhu, Jianyu Wang, Fei Da, Wei Zhang, Qiaohui Gao, Juan Guo, Xia Miao, Junye Liu

**Affiliations:** ^1^ Key Laboratory of Environmental, Health Hazard Assessment and Protection of Shaanxi Province Key Laboratory of Free Radical Biology and Medicine of Shaanxi Province Xi'an China; ^2^ Military Medical Innovation Center Fourth Military Medical University Xi'an China

**Keywords:** Berberine, bile acids, FXR, intestinal regeneration, radiation

## Abstract

Radiation‐induced intestinal injury (RIII) is among the most common complications of radiotherapy in patients with abdominaltumours. At present, there are no effective methods for reducing the occurrence or severity of RIII. Berberine (BBR) is a quaternary ammonium alkaloid extracted from *Coptis chinensis* that has antioxidant, anti‐inflammatory, and protective effects on the intestine. A RIII model was established using 10 Gy of X‐ray total abdominal irradiation (TAI). The effects of BBR on mice exposed to 10 Gy of X‐ray TAI were determined by analysing pathological sections of the mouse intestine. TUNEL staining was used to detect apoptosis of intestinal epithelial cells in mice. Immunohistochemistry was applied to detect the expression of proliferation indicators PCNA and Ki‐67, and immunofluorescence staining was used to quantify BrdU‐positive proliferative cells. BBR stimulates crypt formation ex vivo after irradiation and upregulates the expression of FXR. The expression of goblet cell and intestinal stem cell markers was quantified by qRT‐PCR. The expression of farnesoid X receptor (FXR) in RIII was detected via qRT‐PCR and Western blot. Additionally, a Western blot was performed to detect the protein levels of NF‐κB, p‐p38, total p38, and β‐catenin, and qRT‐PCR was used to measure the mRNA expression levels of pro‐inflammatory factors TNF‐α, IL‐1β, and IL‐6. BBR alleviated RIII, mainly manifested as body weight loss, longer colons, greater numbers of villi, and greater numbers of crypts. BBR also maintained the regeneration ability and promoted the proliferation of crypt cells, reduced the apoptosis rate, and alleviated intestinal injury. Importantly, BBR rescued radiation‐induced dysregulation of these key signalling proteins and pro‐inflammatory factors. BBR failed to promote the repair of RIII when FXR was inhibited. BBR treatment increased the expression of FXR in crypts and was at least protective against radiation‐induced intestinal damage in mice through the modulation of FXR. BBR may be a potential drug for the treatment of radiation‐induced intestinal damage.

## 1 Introduction

Radiotherapy is important for the treatment of multiple tumours and abdominal and pelvic malignancies. The intestine is extremely sensitive to radiation and is inevitably exposed to radiation during radiation therapy [1, 2]. Reports have indicated that 90% of patients who receive abdominal radiation therapy develop radiation‐induced intestinal injury (RIII) within a few weeks [3, 4, 5]. The main symptoms of radiation‐induced intestinal damage include anorexia, vomiting,diarrhoea, dehydration, systemic infection, and in extreme cases, septic shock and even death. RIII severely affects the treatment of patients with abdominal or pelvic tumours and significantly affects their quality of life [6]. Therefore, effective prevention and treatment of radiation enteritis is urgently needed.

Berberine (BBR) is a quaternary ammonium alkaloid extracted from *Coptis chinensis* that has antioxidant, anti‐inflammatory, and protective effects on the intestinal epithelial barrier [7, 8, 9] and is a candidate drug for the treatment of inflammatory bowel disease [10, 11]. Studies have shown that BBR can alter the composition of the gut microbiota and the concentration of its metabolite bile acid [12, 13, 14]; therefore, BBR has long been widely used to treat diarrhoea and intestinal inflammation. Studies have also shown that BBR improves indicators of dextran sulfate sodium (DSS)‐induced ulcerative colitis (UC), such as weight loss, colon shortening, and the inflammatory response. It has also been verified in clinical applications [15]. It is still unclear whether BBR promotes the repair of RIII through bile acid metabolism.

Farnesoid X receptor (FXR, NR1H4), a nuclear receptor critical for bile acid homeostasis, has emerged as a key regulator of intestinal homeostasis [16]. FXR is highly expressed in intestinal epithelial cells (IECs), where it modulates epithelial barrier function, inflammatory responses, and oxidative stress resistance [17, 18]. Preclinical studies have demonstrated that FXR activation protects against intestinal injury in models of inflammatory bowel disease (IBD) and ischemia–reperfusion injury by increasing the expression of tight junction proteins (e.g., ZO‐1 and occludin), suppressing the production of proinflammatory cytokines (e.g., TNF‐α and IL‐6), and activating antioxidant signalling pathways [19].

Recent evidence has indicated that BBR can act as an FXR agonist, modulating bile acid metabolism and improving metabolic disorders in preclinical models [20]. Studies have shown that BBR can reduce inflammation induced by DSS in rats [21, 22, 23]. BBR markedly affects the bioavailability of intestinal nutrients, suggesting its potential influence on the intestinal microbiota [23]. However, whether BBR exerts protective effects against RIII through FXR activation remains unexplored.

Given the critical role of FXR in intestinal epithelial protection and the pharmacological properties of BBR, we hypothesised that BBR‐mediated FXR activation could attenuate radiation‐induced intestinal damage by suppressing inflammation, maintaining the regeneration and proliferation of crypt cells, reducing the apoptosis rate, and alleviating intestinal injury. In this study, we aimed to investigate the protective effects of BBR in a murine model of RIII and elucidate the underlying molecular mechanisms involving FXRsignalling. Our findings may provide novel insights into the therapeutic potential of BBR as an FXR agonist for preventing and treating RIII.

## 2 Materials and Methods

### 2.1 Animals

SPF‐grade male C57BL/6J mice aged 6–8 weeks (body weight: 18–22 g) were obtained from the Laboratory Animal Center of the Fourth Military Medical University (Xi'an, China). Animal experiments were conducted at the university's animal facility, where the mice were housed in ventilated cages under controlled environmental conditions: 22°C–25°C, a 12‐h light/dark cycle, and unrestricted access to food and water. All procedures were approved by the Institutional Ethics Committee of the Fourth Military Medical University (Approval No. 20220743). Euthanasia was performed via intraperitoneal injection of 1% sodium pentobarbital at a dose of 50 mg/kg body weight.

### 2.2 Total Abdominal Irradiation (TAI) and Treatment

Followinganaesthesia, a 3 cm abdominal region (from the sternal xiphoid process to the symphysis pubis) was targeted for radiation exposure, while the remaining body was shielded using 5‐cm‐thick lead blocks. In this study, a Rad Source RS 2000 series X‐ray irradiator (RadSource, USA) was used with an operating voltage of 160 kV, an operating current of 25 mA and a dose rate of 1.032 Gy/min (monitored by the radiation dosimeter in real time). (1) In the control group, mice were orally administered 0.5% sodium carboxymethyl cellulose (CMC) (C8220; Solarbio). (2) In the TAI group, mice were treated with 0.5% CMC and exposed to 10 Gy of total abdominal irradiation. (3) In the TAI + BBR group, mice were treated orally with BBR (25 or 50 mg/kg) [24, 25], which was dissolved in 0.5% of CMC (Selleck; S2271) and given by gavage two hours before irradiation and once a day for 5 days.

### 2.3 BrdU Proliferation Assay

The mice were intraperitoneally injected with BrdU solution (100 mg/kg). After 3 h, the mice were sacrificed, and intestinal tissues were removed and fixed in 4% paraformaldehyde. The proliferation of intestinal crypt cells was detected by immunofluorescence (anti‐BrdU antibody detection). The antibody panel included antibodies against BrdU (Abcam, 1:300). After the samples were washed with PBS, they were incubated with antibodies at 4°C overnight. The sections were photographed with a microscope.

### 2.4 Haematoxylin–Eosin (HE) Staining and Immunohistochemistry (IHC)

After 5 days of irradiation, the intestinal tissue was immediately fixed with 4% paraformaldehyde and then dehydrated with xylene and gradient ethanol. HE staining was followed by embedding the slices (4 μm) according to the manufacturer's protocol. The length of the villi and the number of crypts were measured using ImageJ software. The tissue sections were subjected to antigen retrieval using 0.2 M sodium citrate solution, followed by natural cooling, washing with PBS, blocking, and subsequent rinsing with PBS, after which they were incubated with primary antibodies at 4°C overnight. The antibody panel included antibodies against PCNA (1:800), Ki67 (1:100), and FXR (1:100). After the cells were washed with PBS, they were incubated with two antibodies at 37°C for 1 h. The positive cells were subsequently identified by a DAB kit. The sections were photographed with a microscope.

### 2.5 Intestinal Organoid Culture

The intestinal tissue of the mice was removed and placed in cold PBS. The intestinal lumen was cut along the longitudinal axis on ice. A 1 mL pipette was used to flush the intestinal lumen with PBS, ensuring that the inner wall of the intestinal lumen was clean. The intestine was subsequently cut into small segments. The PBS was discarded, and the intestinal fragments were transferred to the dissociation reagent, containing the reagent and lysed on a shaker for 20 min (200 rpm). On a laminar flow bench, the vortexed intestinal suspension was passed through a 70 μm filter screen and centrifuged at 300 rpm for 5 min; the supernatant was discarded, the sample was resuspended in PBS, and the sample was subsequently centrifuged at 200 rpm for 5 min (this step was repeated twice). The precipitate was collected and placed on ice. The precipitate was resuspended in Intesti Cult Organoid Growth Medium, after which the matrix gel was added at a ratio of 1:1. The sample was gently pipetted and mixed, and then 40 μL was added to the centre of a 24‐well plate such that the number of pits in each drop was 250. The plates or culture dishes were incubated in a 37°C cell incubator for 30 min, supplemented with 500 μL of organoid culture medium, and transferred to the incubator for continued culture. The medium was changed every three days, and the growth status of the intestinal organoids was observed. One week later, the organoids were subcultured and inoculated into 24‐well plates for irradiation at a dose of 4 Gy (1.082 Gy/min). BBR was administered at a dose of 10 μM [26]. The growth of the organoids was observed on the 7th day. Total RNA and protein were isolated concurrently from organoids for the detection of FXR expression.

### 2.6 TUNEL Assay

The intestinal tissue sections were stained using a Roche TUNEL kit following the manufacturer's protocol. Stained samples were observed via confocal microscopy, and statistical analysis of the apoptosis of intestinal epithelial cells was performed.

### 2.7 Quantitative Real‐Time PCR (RT–qPCR)

Total RNA was isolated from fresh intestinal tissues using TRIzol reagent (Invitrogen, USA). cDNA synthesis was conducted with a reverse transcription kit (Abm, USA) following the manufacturer's protocol. Quantitative real‐time PCR (RT–qPCR) was carried out according to the instructions provided by the reagent supplier (Abm, USA). Gene transcription levels were normalised to those of the housekeeping gene GAPDH, and relative mRNA expression was calculated using the 2‐^ΔΔCt^ method. The primer sequences utilised in this study are detailed in Table 1.

**TABLE 1:** Real‐time RT‐PCR primer sequences used in experiments.

Primer	Forward (5′‐3′)	Reverse (5′‐3′)
FXR	GCAACCAGTCATGTACAGATTC	TTATTGAAAATCTCCGCCGAAC
Olfm4	CCTTAGCATTCGCCGCCAGATC	GTTCACCACGCCACCATGACTAC
Lgr5	TTTTTCTGTCAAGTGCTCTTCG	CAGGACACATAGCAAAACGATC
Bmi1	AATGAAGATGAGTCACCAGAGG	CAAAGGAAGATTGGTGGTTAGC
Lrig1	CGCAGCCTAAACCTGAGTTATA	CATTGCTGTTGAGGTACACTTC
Hopx	TGGAGTACAACTTCAACAAGGT	CTAGTCCGTAACAGATCTGCAT
DCLK	CGAACTCTCTCGGATAATGTGA	TGTACTCCAGCTTCTTAAAGGG
Alpi	GAGGTCTTCTCAGTGATGTACC	CATCTCTGCATCTGAGTACCAA
Muc2	CGAGCACATCACCTACCACATCATC	TCCAGAATCCAGCCAGCCAGTC
Tff3	CTCTGGGATAGCTGCAGATTAC	TCAAAATGTGCATTCTGTCTCC
TNF‐α	ACGCTCTTCTGTCTACTGAACTTCG	TGGTTTGTGAGTGTGAGGGTCTG
IL‐6	TTCTTGGGACTGATGCTGGTGAC	GTGGTATCCTCTGTGAAGTCTCCTC
IL‐1β	CTCGCAGCAGCACATCAACAAG	CCACGGGAAAGACACAGGTAGC
GAPDH	TGTTCCTACCCCCAATGTGT	TGTGAGGGAGATGCTCAGTG

### 2.8 Western Blotting

Total intestinal protein was added to the lysate containing protease inhibitor (2%) and phosphatase inhibitor (2%) to make a 10% homogenate, which was subsequently centrifuged at 12,000 rpm for 3 min, after which the supernatant was stored at −80°C, and then the protein concentration was determined by the BCA method. The proteins were separated on a 10% SDS–PAGE gel and then transferred to PVDF membranes (Bio‐Rad Laboratories, USA). The nonspecific binding site on the membrane was blocked with 5% skim milk powder in Tris buffer/Tween‐20 (TBST) for 2 h at room temperature and then incubated (overnight, 4°C) with different primary antibodies (1:1000), namely, PCNA (CST # 13110), FXR (Invitrogen # A9033A), NF‐κB (CST # 8242), p‐p38 (CST # 4511), p38 (CST # 9212), β‐catenin (CST # 8480) and β‐actin (CST # 8H10D10). The membrane was incubated with the secondary antibody (1:5000) for 2 h and then washed with TBST 5 times. The protein signal was detected by an enhanced chemiluminescence (ECL) lead reagent. Protein strength was analysed by ImageJ software.

### 2.9 Periodic Acid–Schiff (PAS) Staining

The slices were routinely dewaxed in water, washed in distilled water, oxidised with periodate solution, and then washed in distilled water, after which the excess water was discarded. The slices were then dyed with Schiff's reagent and rinsed with running water to remove excess stain. Haematoxylin was used to nucleate the sample, which was then rinsed under running water and reverse blue, after which the excess water was shaken off. Conventional stepwise ethanol dehydration, clearing with xylene, and sealing with neutral gum were performed.

### 2.10 Statistical Analysis

Statistical analyses were conducted using GraphPad Prism 9.2 (San Diego, CA, USA). Data were analysed via an unpaired Student's *t*‐test or one‐way ANOVA followed by Tukey's post hoc multiple comparisons test, as appropriate. All measurements are presented as the mean ± SD. Statistical significance was defined as *p* < 0.05.

## 3 Results

### 3.1 BBR Protected Against Radiation‐Induced Intestinal Injury in Mice

**FIGURE 1:**
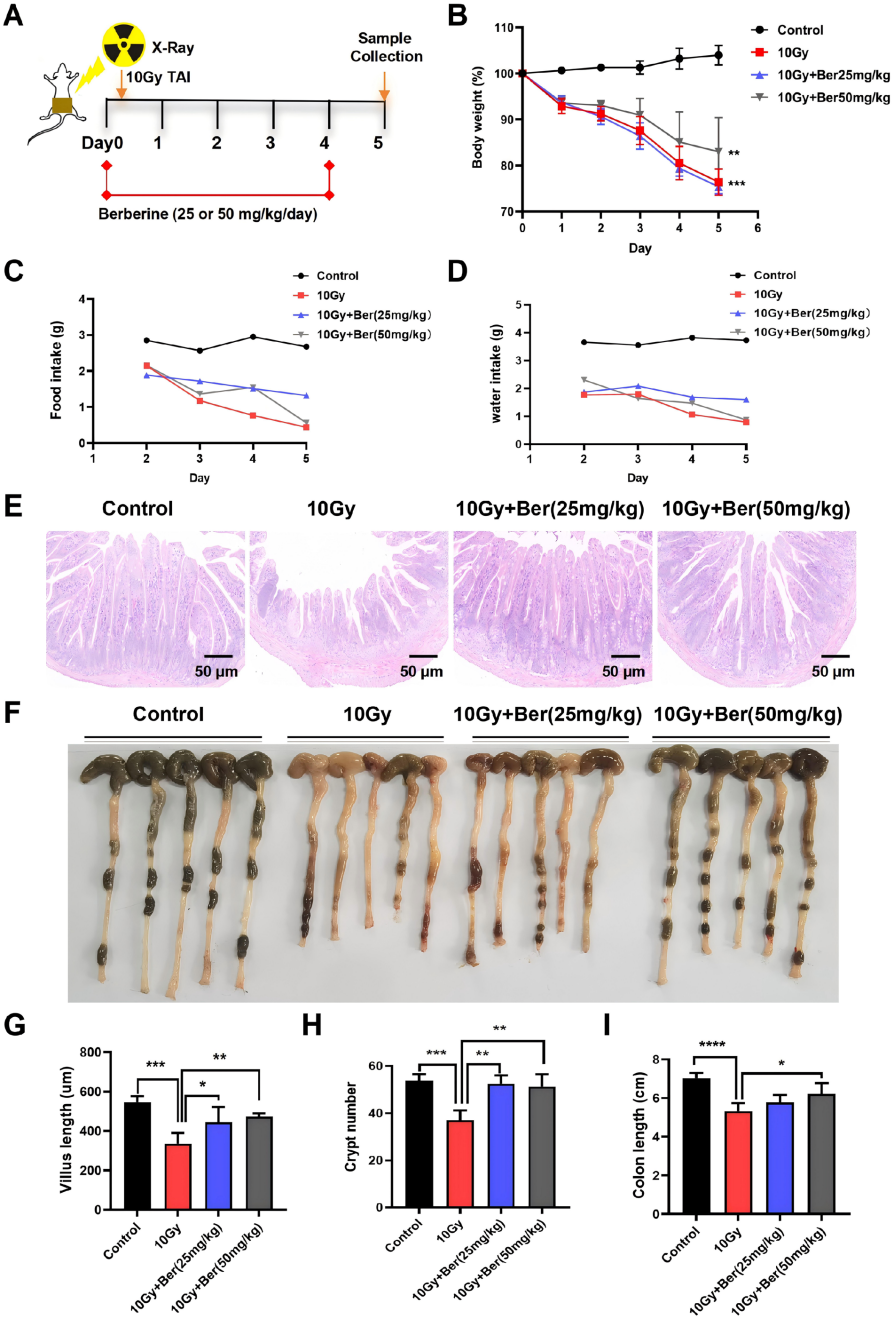
The administration of BBR alleviated RIII in mice. (A) Schematic diagram of the experimental design. (B) Effects of BBR administration on the body weight of mice with RIII, the data are expressed as the mean ± SD (*n* = 10). (C) Effects of BBR administration on the average food intake of mice. (D) Effects of BBR administration on the average water intake of mice. (E) Haematoxylin–eosin (HE) staining of the intestine, scale bar, 50 μm. (F) The morphology of the colon in each group of mice. (G) Quantification of the villus length of the mice. (H) Quantification of crypt numbers in the mice. (I) Quantification of colon length in the mice. Villus length and crypt numbers were quantified in at least 5 fields of view per mouse. The data are presented as the mean ± SD. *p < 0.05, **p < 0.01, ***p < 0.001, ****p < 0.0001, one‐way ANOVA followed by Tukey's multiple comparisons post hoc test.

To evaluate the effect of BBR on RIII, we established a mouse model for total abdominal radiation (TAI), in which the mice were irradiated with 10 Gy of X‐ray radiation at a rate of 1.032 Gy/min and orally administered BBR (Figure 1A). The mice in the IR group had the fastest weight loss, and this decline improved when the mice were given BBR (Figure 1B). On the third day after irradiation, the average cumulative food intake and water intake of BBR‐treated mice were significantly greater than those of irradiated mice (Figure 1C,D). HE staining was performed to examine the intestinal tissue structure, and we found that BBR also alleviated structural damage to the intestine (Figure 1E). The colon length of the BBR‐treated mice was significantly longer than that of the irradiated mice (Figure 1F). Compared with those of irradiated mice, the villus length and crypt number of the mice treated with BBR improved significantly (Figure 1G,H). Meanwhile, we also found that BBR has the same protective effect in female mice as that in male mice (Figure S1). No histological abnormalities or morphometric changes were detected in the intestine, liver and kidney following BBR administration (Figure S2), confirming the safety of these doses for further in vivo studies. These findings suggest that BBR can promote the integrity of the intestinal epithelium and reduce intestinal injury caused by radiation.

### 3.2 BBR Promoted Intestinal Cell Proliferation and Alleviated Apoptosis of Mouse Intestinal Epithelial Cells After Irradiation

**FIGURE 2:**
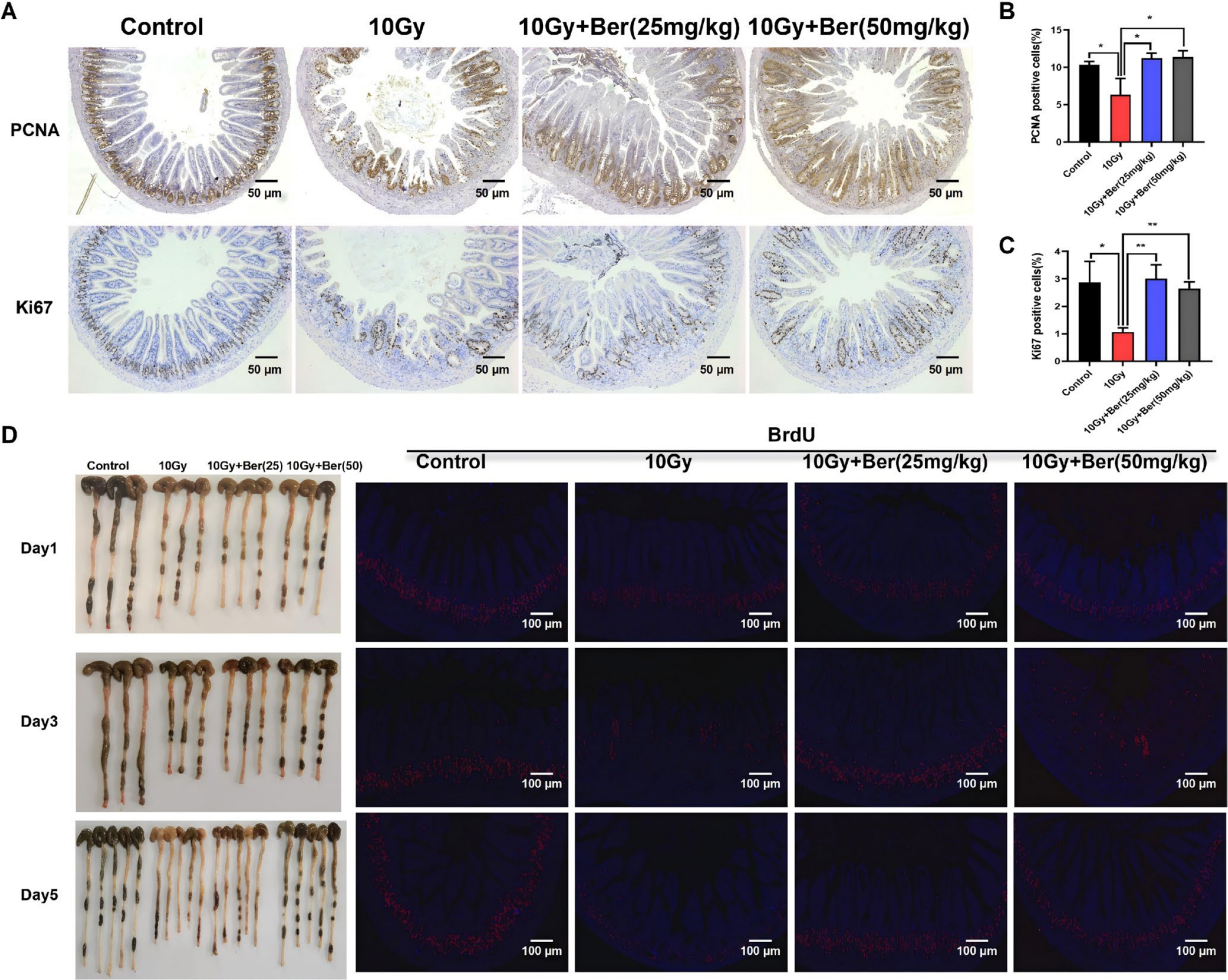
BBR promoted intestinal proliferation after irradiation. Improvement in the proliferation ability of intestinal epithelial cells in C57BL/6J mice treated orally with BBR or TAI was observed. (A) Immunohistochemical staining of PCNA and Ki67 in mouse intestinal crypts. The percentage of PCNA (B) and Ki67 (C) positive cells per crypt of five crypts was analysed for each mouse. (D) Gross colonic morphology and BrdU immunofluorescence staining of mice with RIII on the first, third and fifth days after irradiation. The data are reported as the mean ± SD (*n* = 5). *p < 0.05, **p < 0.01, one‐way ANOVA followed by Tukey's multiple comparisons post hoc test.

**FIGURE 3:**
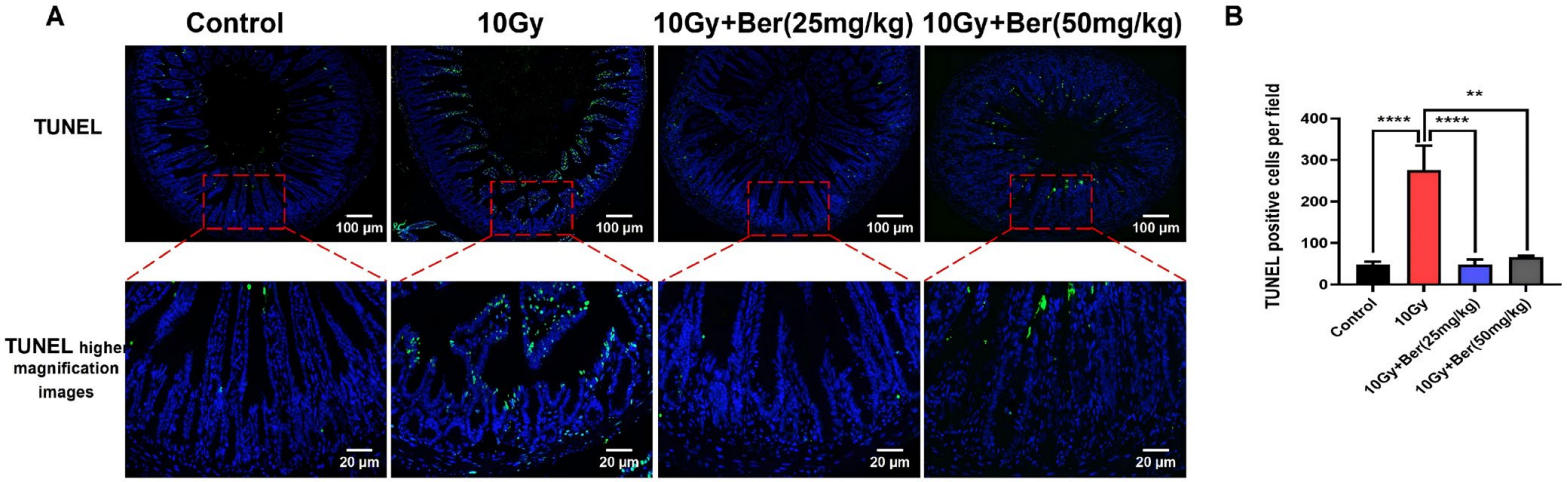
BBR alleviated apoptosis of mouse intestinal epithelial cells after irradiation. (A) Apoptosis in the intestine was analysed by TUNEL staining. Scale bar, 100 μm (above), and scale bar = 20 μm (below). (B) The number of TUNEL‐positive cells per field of view was determined. The data are reported as the mean ± SD (*n* = 3). **p < 0.01, ****p < 0.0001, one‐way ANOVA followed by Tukey's multiple comparisons post hoc test.

The synthesis and expression of PCNA are considered important markers of cell proliferation. The Ki67 protein is expressed throughout every cell cycle and promotes cell proliferation, indicating the ability of cells to proliferate. Next, we attempted to determine whether BBR can alleviate the destruction of intestinal IECs after irradiation. Immunohistochemical staining and analysis revealed that BBR supplementation promoted intestinal epithelial cell proliferation (Figure 2A). Moreover, our results revealed that BBR increased the expression of the proliferation indices PCNA and Ki67 (Figure 2B,C). BrdU immunofluorescence staining was performed on the intestinal tissues of mice on the 1st, 3rd, and 5th days after irradiation. The results showed that BBR promoted cell proliferation in mice with RIII (Figure 2D). IHC analysis of PCNA and Ki67 showed equivalent levels of intestinal epithelial proliferation across all groups, confirming that BBR fails to drive proliferation of intestinal epithelial cells in normal mice (Figure S3). These findings suggest that BBR can promote cell proliferation in the mouse intestinal tract after radiation injury. To study the effect of BBR on intestinal cells apoptosis in mice, TUNEL staining was used to detect changes in apoptotic cells (Figure 3A). Compared with that in the control group, the number of apoptotic cells in the TAI‐treated group increased, whereas the number of apoptotic cells in the BBR‐treated group significantly decreased (Figure 3B).

### 3.3 BBR Stimulates Crypt Formation Ex Vivo After Irradiation and Upregulates the Expression of FXR

**FIGURE 4:**
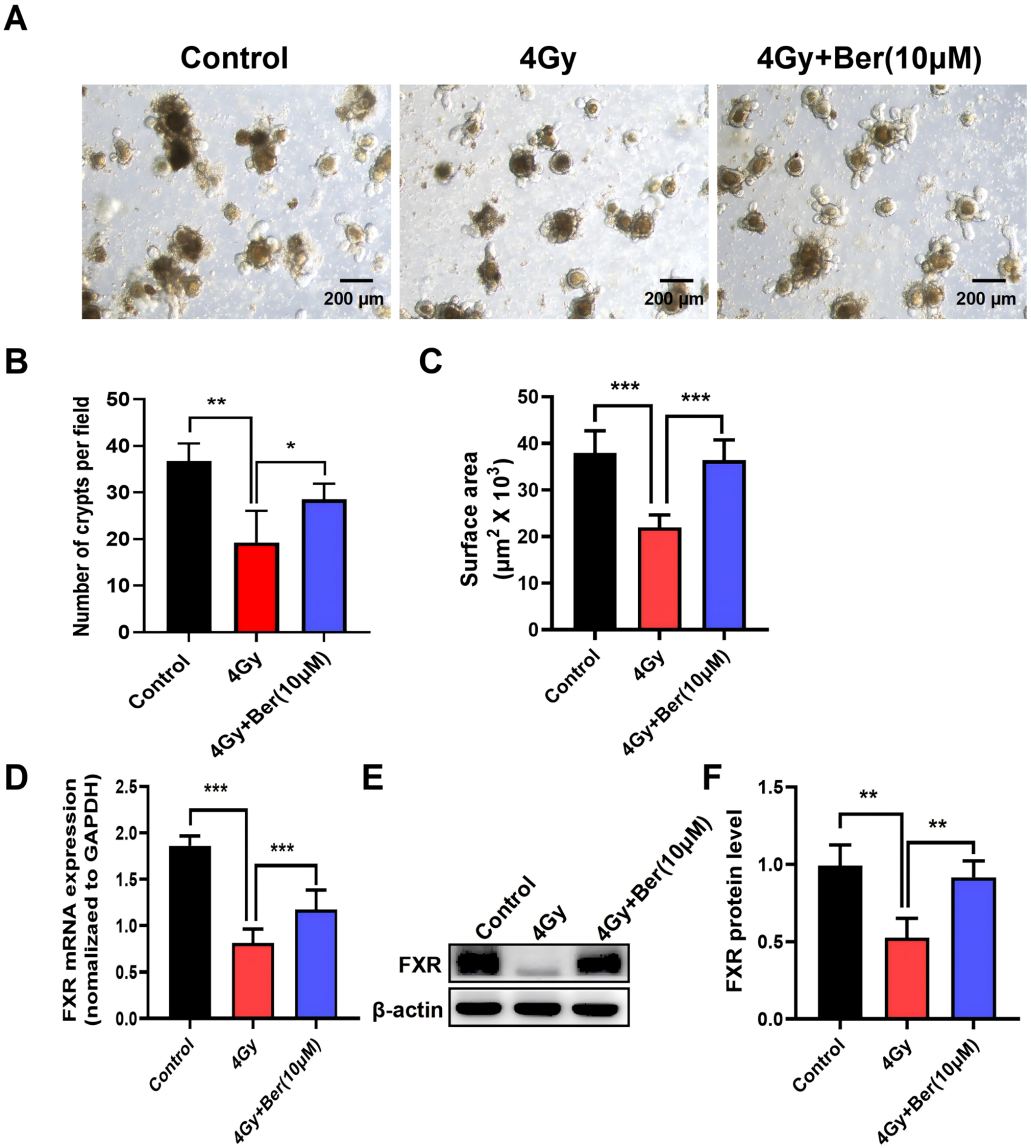
BBR promoted the proliferation of organoids after irradiation and upregulated the expression of FXR. (A) Representative images of intestinal organoids treated with berberine (10 μM) on Day 7 after 4 Gy of X‐ray irradiation. Scale bars, 200 μm. (B) The number of newborn crypts was quantified for statistical analysis. (C) Quantification of total crypt surface area of primary enteroids per microscopic field. (D) qRT‐PCR analysis of FXR mRNA abundance. (E, F) Western blot representative bands and quantitative densitometry of FXR protein. The data are presented as the mean ± SD. *p < 0.05, **p < 0.01, ***p < 0.001, one‐way ANOVA followed by Tukey's multiple comparisons post hoc test.

To further clarify the protective effects of BBR, intestinal organoids were also used in our study. After exposure to 4 Gy of X‐ray radiation, BBR promoted intestinal organoid growth (Figure 4A,B). Radiation at 4 Gy can significantly reduce the total surface area of primary intestinal organoids. Combined treatment with 10 μM BBR can significantly reverse the reduction in the surface area of organoids caused by radiation, and the size of organoids can be restored to a level close to that of the normal control group (Figure 4C). The above results suggest that 4 Gy ionising radiation can severely damage the growth ability and structural integrity of intestinal primary organoids, while 10 μmol/L BBR can effectively antagonise radiation damage and maintain the proliferation activity and structural homeostasis of intestinal organoids. Radiation markedly decreased FXR mRNA and protein abundance, whereas BBR reversed this repression (Figure 4D–F). Overall, BBR mitigates radiation‐triggered intestinal organoid injury along with FXR recovery.

### 3.4 BBR Increased the Number of Goblet Cells and the Expression of Stem Cell Markers in Mice With RIII

**FIGURE 5:**
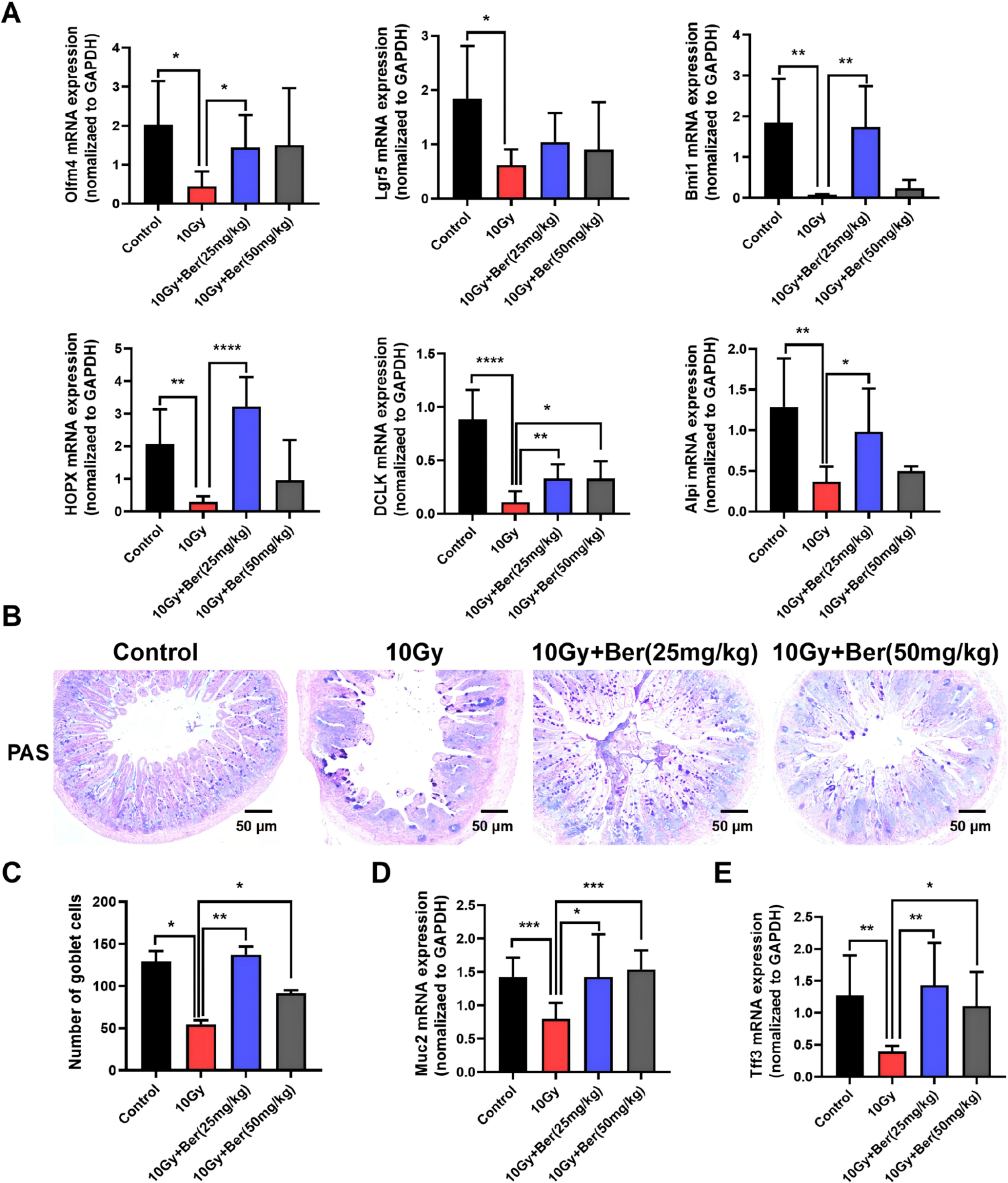
BBR increased the number of goblet cells and the expression of stem cell markers in mice with RIII. To determine whether BBR affects the loss of intestinal stem cells, after irradiation, qRT–PCR was used to detect the expression of different types of intestinal stem cell markers. (A) The expression of Olfm4 and Lgr5 stem cell markers in the basal part of the crypts, the expression of the “+4” intestinal stem cell markers Bmi1 and HOPX, and the expression of the intestinal stem cell‐related markers DCLK and Alpi were measured by qRT–PCR. (B) PAS staining of intestinal goblet cells. (C) The number of goblet cells per field of view was determined. (D, E) The expression of the goblet cell markers Muc2 and Tff3 in the small intestine of mice was measured by qRT–PCR. The results are expressed as the mean ± SD. *n* = 8 mice per group. *p < 0.05, **p < 0.01, ***p < 0.001, ****p < 0.0001, unpaired Student's t test.

Intestinal stem cells are located at the bottom of the intestinal recess and maintain the self‐renewal of intestinal epithelial cells. To investigate the ability of BBR to enhance intestinal cell regeneration, we measured the expression of intestinal stem cell markers in mouse intestinal crypt cells, including those of basal crypt stem cells, “+4” intestinal stem cells and intestinal stem cells in the villi region of the intestine (Figure 5A). The results showed that BBR promoted the growth of intestinal stem cells in the crypt base, “+4” intestinal stem cells and intestinal villi. These results suggest that BBR can promote intestinal stem cell renewal and improve the regenerative ability of ISCs in irradiated mice.

Goblet cells are columnar epithelial cells that secrete mucin and have a barrier function of protecting the intestinal epithelium. Damage to the mucus layer results in heightened intestinal permeability, which is then accompanied by bacterial translocation and systemic inflammatory reactions. To study the effect of BBR on goblet cells after intestinal damage caused by irradiation, PAS staining was performed on irradiated intestines (Figure 5B). As shown in Figure 3B, compared with that in the control group, the number of goblet cells stained by PAS in the IR group was significantly lower. Compared with those in the IR group, the expression of PAS‐stained neutral mucin and the number of goblet cells were greater in the IR group treated with BBR (Figure 5B,C). Mucin 2 (Muc2) is a differentiated goblet cell marker that is an important component of the intestinal mucosal barrier [27, 28]. In addition, the mRNA levels of Muc2 and trilobal factor 3 (Tff3), another marker of goblet cell differentiation, were lower in the IR group than in the control group but significantly increased in the BBR‐treated IR group (Figure 5D,E). These results suggest that BBR can reduce intestinal injury caused by radiation, promote the recovery of goblet cells and protect the intestinal epithelium.

We further explored the underlying molecular mechanisms via western blot detection of key signalling molecules. Immunoblotting results revealed that ionising radiation notably suppressed the expression of NF‐κB, p‐p38, and β‐catenin, while total p38 protein level remained stable. BBR intervention dose‐dependently restored the downregulated expression of these proteins (Figure S4).

### 3.5 BBR Activated Intestinal Farnesoid X Receptor Signalling in Irradiated Mice

**FIGURE 6:**
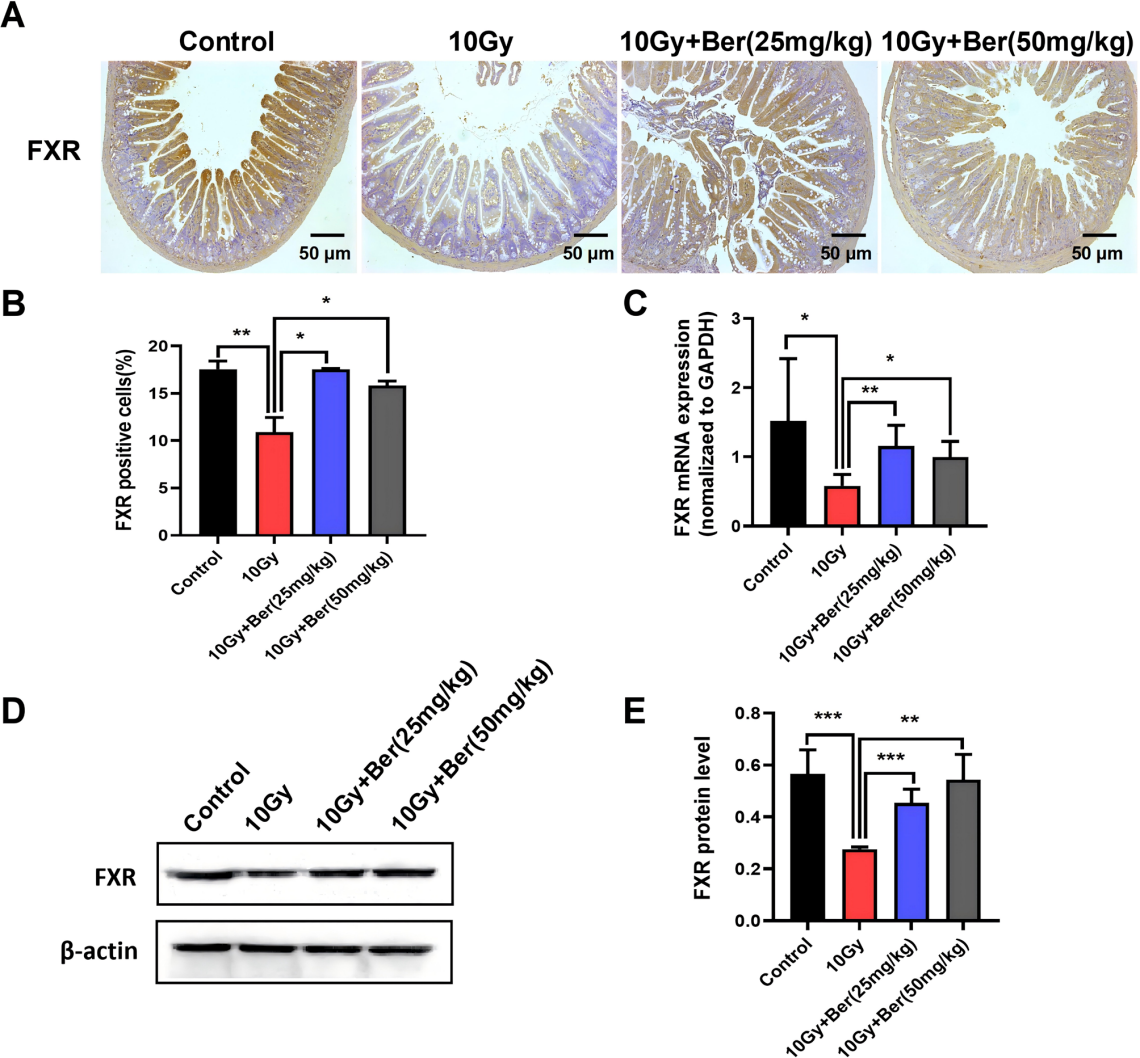
FXR expression was enhanced in the crypt cells of irradiated mice treated with BBR. (A) Representative images of immunohistochemical staining for FXR in the intestines of normal mice, irradiated mice and BBR‐treated mice. (B) Quantitative analysis of FXR‐positive cells in the intestine by immunohistochemical staining. (C) The mRNA expression of FXR in intestinal crypt cells was measured by qRT–PCR. (D, E) The protein levels of FXR in the mouse enteroids were analysed by western blotting and quantified by densitometry. β‐Actin was used as a loading control. The data represent 3 independent experiments. The results are expressed as the mean ± SD. *n* = 5 mice per group. *p < 0.05, **p < 0.01, ***p < 0.001, one‐way ANOVA followed by Tukey's multiple comparisons post hoc test.

Some BA species, such as deoxycholic acid (DCA) and ursodeoxycholic acid (UDCA), which are regulated by BBR, are recognised as FXR agonists. Therefore, we subsequently investigated the protein expression of intestinal FXR in mice with RIII that were treated with BBR. To understand the underlying mechanism through which BBR facilitates intestinal regeneration postirradiation, we measured the expression levels of FXR. Immunohistochemical staining revealed that BBR treatment significantly increased the FXR expression level (Figure 6A), accompanied by an increase in the number of FXR‐positive cells (Figure 6B). Consistent with these results, BBR treatment increased FXR mRNA expression levels in crypt cells (Figure 6C). In addition, western blotting analysis revealed that BBR increased the protein expression level of FXR (Figure 6D,E).

### 3.6 BBR Was Unable to Promote the Repair of RIII After Inhibiting FXR

**FIGURE 7:**
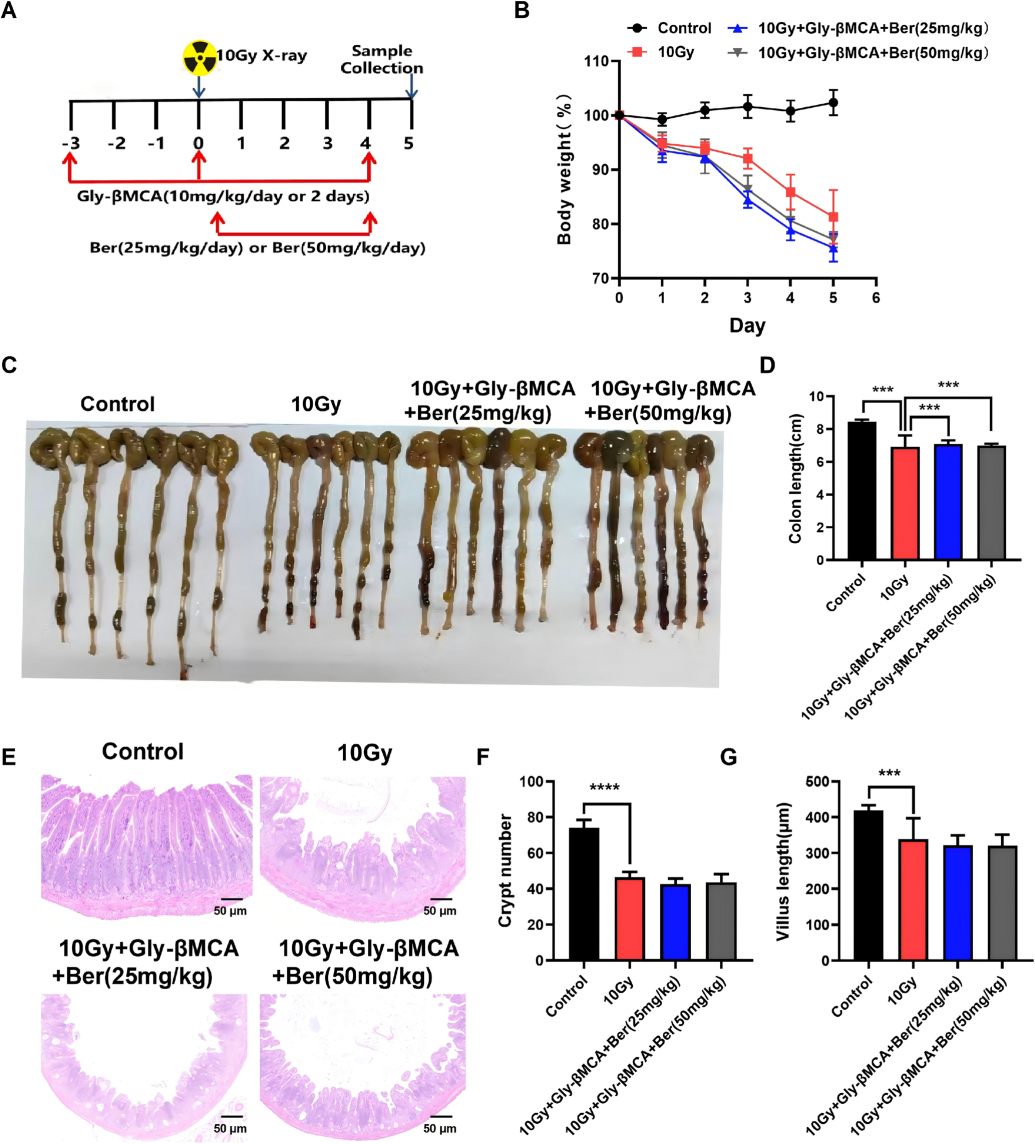
BBR did not promote the repair of RIII after inhibition of FXR expression. (A) Schematic diagram of the effects of an FXR inhibitor and BBR on RIII. (B) Effects of BBR administration on the body weight of mice with RIII after treatment with the FXR inhibitor gly‐βMCA; the data are expressed as the mean ± SD (*n* = 8). (C) The morphology of the colon in each group of mice. (D) Quantification of colon length in the mice. (E) Haematoxylin–eosin (HE) staining of the intestine; scale bar, 50 μm. (F) Quantification of crypt numbers in the mice. (G) Quantification of the villus length of the mice. Crypt numbers and villus length were quantified in at least 5 fields of view per mouse. The data are presented as the mean ± SD. ***p < 0.001, ****p < 0.0001, one‐way ANOVA followed by Tukey's multiple comparisons post hoc test.

To further determine whether FXR intervention is necessary for the potential mechanism through which BBR promotes the repair of RIII in mice, the FXR inhibitor gly‐βMCA was used. to intervene. The mice were intragastrically administered gly‐βMCA (10 mg/kg) every day for 4 days before irradiation. On the 4th day, the abdomen of the mice was intragastrically irradiated with 10 Gy of X‐ray radiation after the administration of the FXR inhibitor. After irradiation, the mice were intragastrically treated with BBR every day, during which time gly‐βMCA (10 mg/kg) was intragastrically administered every 2 days (Figure 7A). Compared with the irradiation group, the irradiated mice treated with two doses of BBR and the FXR inhibitor had more obvious weight loss (Figure 7B), and the degree of injury increased. Compared with those of normal mice, the colon length was shortened (Figure 7C,D), the number of crypts was lower, and the length of the villi of the intestinal tissue was shortened (Figure 7E–G). The experimental findings indicated that following the inhibition of FXR, BBR failed to alleviate c in mice.

### 3.7 BBR Did Not Promote Intestinal Proliferation or Alleviate Apoptosis in Mouse Intestinal Epithelial Cells After Inhibition of FXR

**FIGURE 8:**
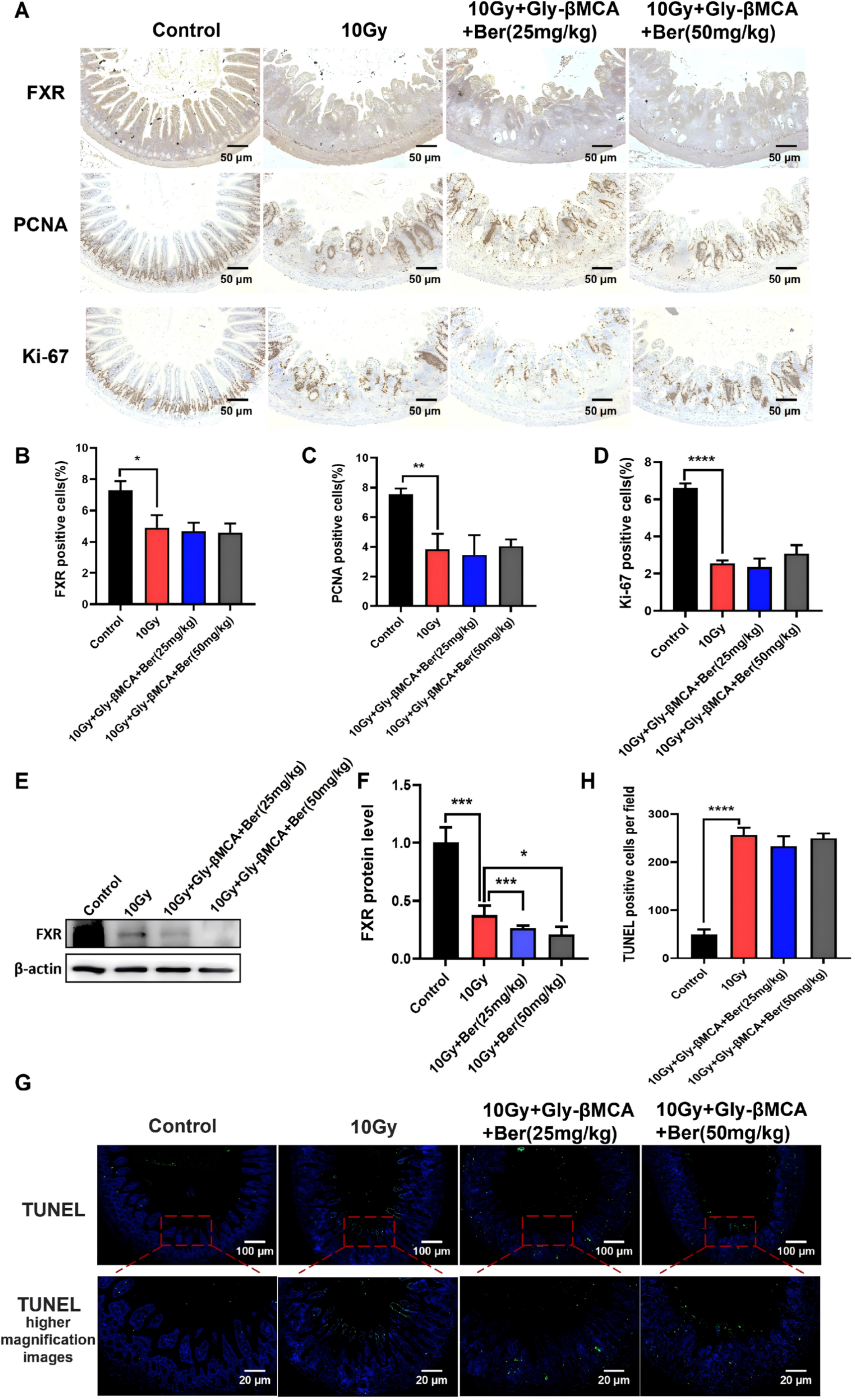
BBR did not promote intestinal proliferation or alleviate apoptosis in mouse intestinal epithelial cells after inhibition of FXR expression. (A) Immunohistochemical staining of FXR, PCNA and Ki67 in mouse intestinal crypts. The percentage of FXR (B), PCNA (C) and Ki67 (D) positive cells per crypt in five crypts was analysed for each mouse. (E, F) The protein levels of FXR in the mouse enteroids were analysed by western blotting and quantified by densitometry. (G) Apoptosis in the intestine was analysed by TUNEL staining. Scale bar, 100 μm (above), and scale bar = 20 μm (below). (H) The number of TUNEL‐positive cells per field of view was determined. The data are reported as the mean ± SD (*n* = 4). *p < 0.05, **p < 0.01, ***p < 0.001, ****p < 0.0001, one‐way ANOVA followed by Tukey's multiple comparisons post hoc test.

Following FXR inhibition, IHC staining was used to measure the expression of FXR, PCNA, and Ki67 in the intestinal tissues of BBR‐treated mice. Compared with that in the normal group, the expression of FXR in the intestinal tissues of mice treated with BBR after FXR inhibition was downregulated (Figure 8A,B). The expression of PCNA and Ki67 was downregulated in the intestinal recess (Figure 8A,C,D). Western blot results demonstrated that pharmacological inhibition of FXR markedly downregulated the protein expression level of FXR (Figure 8E,F). The results showed that BBR could not promote the proliferation of intestinal crypt cells after FXR inhibition. TUNEL staining was used to detect apoptosis in mouse intestinal epithelial cells (Figure 8G). The results revealed that the number of intestinal epithelial cells undergoing apoptosis was not lower in mice with RIII treated with BBR and the FXR inhibitor than in those treated with irradiation alone (Figure 8H). Therefore, BBR could not reduce the apoptosis of intestinal epithelial cells in mice with RIII after inhibiting FXR.

## 4 Discussion

The intestine is highly sensitive to ionising radiation, serving as the primary target of damage in abdominal radiotherapy and environmental overexposure. RIII significantly compromises the efficacy of tumour radiotherapy. It is the main dose‐limiting factor of abdominal and pelvic radiotherapy, but there is currently no effective prevention or treatment method [29, 30]. Identifying novel protective agents and therapeutic targets to mitigate radiation‐induced intestinal damage is therefore of urgent clinical importance. While previous studies have explored the potential of herbal medicines and extracts to reduce radiation toxicity, research on drugs that protect against intestinal damage caused by IR is still needed [31]. In this study, we demonstrate that berberine (BBR), a bioactive alkaloid derived from *Coptis chinensis*, exerts protective effects against radiation‐induced intestinal damage.

Coptis has a long history of use in treating enteritis with confirmed efficacy, and BBR is its principal active component and is known for its anti‐inflammatory, antibacterial, and antitumor properties [32, 33]. Biosafety of 25/50 mg/kg BBR was pre‐validated in normal mice: No organ pathology or altered intestinal morphology, and no aberrant epithelial proliferation. These data ruled out confounding BBR‐induced toxicity or dysplasia, confirming dose safety for radioprotection assays. Radiation‐induced gastrointestinal syndrome typically manifests as weight loss and reduced food intake. Our experimental data revealed that compared with control mice, mice treated with BBR starting two hours before abdominal irradiation maintained significantly greater body weight, water intake, and food intake, indicating that BBR supports metabolic homeostasis during radiation exposure. Histological analysis revealed that radiation often causes villus blunting, fusion, and crypt depletion, disrupting epithelial integrity and homeostasis [34]. Notably, BBR treatment preserved the architecture of intestinal crypts and villi in mice subjected to 10 Gy of total abdominal irradiation (TAI), suggesting the maintenance of epithelial structural integrity.

Radiation‐induced tissue damage is characterised by increased apoptosis, a process that is observed to be significantly attenuated by BBR, as evidenced by reduced numbers of TUNEL‐positive cells in the intestinal epithelium. These findings suggest that BBR protects against radiation injury by inhibiting epithelial cell apoptosis. Additionally, cell proliferation is critical for postradiation tissue repair [35]. Markers of cell proliferation, such as PCNA and Ki67, were upregulated in BBR‐treated mice, indicating the enhanced regenerative capacity of intestinal epithelial cells following radiation injury [36]. In vitro organoid assays validated the direct radioprotective action of BBR on intestinal epithelial cells. BBR rescued radiation‐caused morphological defects of enteroids, eliminating interference from gut microbiota and systemic immunity.

Intestinal epithelial stem cells (ISCs) are essential for maintaining intestinal homeostasis and facilitating repair after damage [37]. At the crypt base, ISCs differentiate into goblet cells, which secrete mucus critical for epithelial regeneration and defence against radiation injury [38]. Mucus secreted by goblet cells plays a critical role in RIII [39, 40]. Our findings show that BBR promotes the regeneration of ISCs and enhances goblet cell secretion in irradiated intestines, suggesting a dual role in stem cell‐mediated repair and mucosal barrier reinforcement.

Mechanistically, our results further elucidated the downstream signalling changes triggered by irradiation and BBR intervention. Ionising radiation markedly suppressed the protein expression of NF‐κB, phosphorylated p38 (p‐p38) and β‐catenin, while total p38 protein expression remained stable, indicating radiation specifically blocked the phosphorylation activation of the p38 MAPK pathway and inhibited NF‐κB and β‐catenin signalling cascades. Dose‐dependent BBR treatment effectively reversed these radiation‐induced molecular abnormalities. The restoration of these key signalling pathways provides a molecular explanation for the recovered epithelial proliferation, crypt regeneration and intestinal structural integrity in irradiated intestinal tissues following BBR treatment.

Previous research has linked BBR to the modulation of bile acid (BA) metabolism and gut microbial function via the FXR, a nuclear receptor vital for BA homeostasis, cholesterol metabolism, and anti‐inflammatory responses [41]. While FXR has been implicated in anti‐inflammatory and antifibrotic pathways [42, 43], its role in BBR‐mediated protection against RIII is unknown. Our experiments revealed that BBR activated intestinal FXR signalling in irradiated mice; importantly, inhibition of FXR abrogated the protective effects of BBR, blocking epithelial cell proliferation and failing to reduce apoptosis. This finding identifies intestinal FXR as a critical mediator of the therapeutic effects of BBR.

Mounting evidence indicates that BBR exerts dual regulatory effects on FXR expression and activation within intestinal epithelial cells. On one hand, BBR remodels the composition of gut microbiota and reshapes the intestinal bile acid pool [44]; the accumulated endogenous bile acids serve as natural ligands to indirectly stimulate FXR activation in IECs. On the other hand, existing literature confirms that BBR can directly upregulate FXR transcription, leading to increased FXR mRNA and protein abundance [20, 45]. Combined with our phenotypic data showing that FXR antagonist only partially offsets the radioprotective benefits of BBR, we infer that BBR alleviates radiation‐induced intestinal damage through two coordinated routes: Indirect activation of FXR signalling mediated by microbiota‐bile acid axis, and direct transcriptional upregulation of FXR in intestinal epithelium. Future research will focus on dissecting the precise molecular cascades governing these two regulatory patterns.

In conclusion, our study established BBR as a protective agent against RIII, primarily through activation of the FXR signalling pathway. These findings highlight FXR as a potential target for BBR‐based therapy and provide novel insights into the translational application of this natural compound for radiation enteropathy, providing effective intervention ideas for further drug development and clinical treatment.

## Author Contributions

**Wei Zhang:** investigation, formal analysis. **Jianyu Wang:** methodology, visualization. **Lei Zhu:** methodology, data curation. **Xia Miao:** software, validation. **Fei Da:** investigation, validation. **Xihao Liu:** methodology, data curation. **Li Guo:** conceptualization, investigation, writing – original draft, writing – review and editing, visualization, methodology, formal analysis, data curation. **Lu Guo:** methodology, data curation, formal analysis, validation. **Qiaohui Gao:** validation, formal analysis. **Junye Liu:** writing – review and editing, visualization, conceptualization, funding acquisition, software, project administration, resources. **Juan Guo:** supervision, formal analysis.

## Funding

We gratefully acknowledge that this work was supported by grants from the National Natural Science Foundation of China (82330100) and the Project funded by China Postdoctoral Science Foundation (GZC20233579).

## Conflicts of Interest

The authors declare no conflicts of interest.

## Supporting information


**Figure S1:** The administration of BBR alleviated RIII in female mice. The mice were divided into 4 groups: Normal mice, 10 Gy X‐ray irradiated mice and mice treated with BBR (25 or 50 mg/kg). (A) Schematic diagram of the experimental design. (B) Effects of BBR administration on the body weight of mice with RIII, the data are expressed as the mean ± SD (*n* = 10). (C) Haematoxylin–eosin (HE) staining of the intestine, scale bar, 50 μm. (D) The morphology of the colon in each group of mice. (E) Quantification of colon length in the mice. (F) Quantification of crypt numbers in the mice. (G) Quantification of the villus length of the mice. Crypt numbers and villus length were quantified in at least 5 fields of view per mouse. The data are presented as the mean ± SD. *p < 0.05, **p < 0.01, ***p < 0.001, ****p < 0.0001, one‐way ANOVA followed by Tukey's multiple comparisons post hoc test.
**Figure S2:** BBR treatment exhibits no obvious toxicity in normal mice. (A) Effects of BBR administration on the body weight of normal mice, the data are expressed as the mean ± SD (*n* = 10). (B) Representative macroscopic images of excised colons from the Control group, Control+Ber (25 mg/kg) group, and Control+Ber (50 mg/kg) group. (C) Statistical quantification of colon length. (D) Haematoxylin and eosin (H&E) staining of intestine, liver, and kidney tissue sections from each experimental group; scale bar = 50 μm. (E) Quantification of intestinal crypt number. (F) Quantification of intestinal villus length. Data are presented as mean ± SD (*n* = 4). No statistically significant differences were detected among the three groups.
**Figure S3:** BBR treatment does not alter intestinal epithelial cell proliferation in normal mice. (A) Representative immunohistochemistry images of PCNA and Ki67 staining in intestinal sections of the Control group, Control+Ber (25 mg/kg) group and Control+Ber (50 mg/kg) group; scale bar = 50 μm. (B) Statistical quantification of the percentage of PCNA‐positive epithelial cells in each group. (C) Statistical quantification of the percentage of Ki67‐positive epithelial cells in each group. Data are presented as mean ± SD (*n* = 4). No significant intergroup differences were observed.
**Figure S4:** BBR rescues radiation‐induced dysregulation of key signalling proteins. (A) Representative immunoblot bands for target proteins; β‐actin was used as the internal reference protein, and the molecular weight of each protein is labelled on the right side of the blots. (B–E) Relative quantitative analysis of NF‐κB (B), p‐p38 (C), total p38 (D) and β‐catenin (E) protein levels normalised to β‐actin. (F–H) Relative mRNA expression levels of pro‐inflammatory factors TNF‐α, IL‐1β and IL‐6 detected by qRT‐PCR, normalised to GAPDH. Data are presented as mean ± SD (*n* = 3). Statistical analysis was performed using one‐way ANOVA followed by Tukey's post hoc multiple comparison test. *p < 0.05, **p < 0.01, ****p < 0.0001.

## Data Availability

The data that support the findings of this study are available from the corresponding author upon reasonable request.
