# Supplementary material for: The Role and Mechanism of Berberine as a Candidate Drug for the Treatment of Radiation‐Induced Intestinal Injury
Source: J Cell Mol Med. 2026 Aug 9;30(15):e71281. doi: 10.1111/jcmm.71281 (PMC13454386; doi:10.1111/jcmm.71281)
Supplement: Supplementary file 1 [file JCMM-30-e71281-s001.zip › jcmm71281-sup-0002-FigureS1-S4.doc]

**Supplementary Material:**

**Supplementary Fig 1.** **The administration of BBR alleviated** **RIII in female mice.** The mice were devided into 4 groups, normal mice, 10 Gy X-ray irradiated mice and mice treated with BBR (25 or 50 mg/kg). **(A)** Schematic diagram of the experimental design. **(B)** Effects of BBR administration on the body weight of mice with RIII, the data are expressed as the mean ± SD (n = 10). **(C)** Hematoxylin‒eosin (HE) staining of the intestine, scale bar, 50 μm. **(D)** The morphology of the colon in each group of mice. **(E)** Quantification of colon length in the mice. **(F)** Quantification of crypt numbers in the mice. **(G)** Quantification of the villus length of the mice. crypt numbers and villus length were quantified in at least 5 fields of view per mouse. The data are presented as the mean ± SD. **p*< 0.05, ***p*< 0.01, ****p*< 0.001, *****p*< 0.0001, one-way ANOVA followed by Tukey’s multiple comparisons post hoc test.

**Supplementary Fig 2. BBR treatment exhibits no obvious toxicity on normal mice. (A)** Effects of BBR administration on the body weight of normal mice, the data are expressed as the mean ± SD (n = 10). **(B)** Representative macroscopic images of excised colons from the Control group, Control+Ber (25 mg/kg) group, and Control+Ber (50 mg/kg) group. **(C)** Statistical quantification of colon length. **(D)** Hematoxylin and eosin (H&E) staining of intestine, liver, and kidney tissue sections from each experimental group; scale bar = 50 μm. **(E)** Quantification of intestinal crypt number; **(F)** Quantification of intestinal villus length. Data are presented as mean ± SD (n = 4). No statistically significant differences were detected among the three groups.

**Supplementary Fig 3. BBR treatment does not alter intestinal epithelial cell proliferation in normal mice. (A)** Representative immunohistochemistry images of PCNA and Ki67 staining in intestinal sections of the Control group, Control+Ber (25 mg/kg) group and Control+Ber (50 mg/kg) group; scale bar = 50 μm. **(B)** Statistical quantification of the percentage of PCNA-positive epithelial cells in each group. **(C)** Statistical quantification of the percentage of Ki67-positive epithelial cells in each group. Data are presented as mean ± SD (n = 4). No significant intergroup differences were observed.

**Supplementary Fig 4. BBR rescues radiation-induced dysregulation of key signaling proteins. (A)** Representative immunoblot bands for target proteins; β-actin was used as the internal reference protein, and the molecular weight of each protein is labeled on the right side of the blots. **(B–E)** Relative quantitative analysis of NF-κB **(B)**, p-p38 **(C)**, total p38 **(D)** and β-catenin **(E)** protein levels normalized to β-actin. (F–H) Relative mRNA expression levels of pro-inflammatory factors TNF-α, IL-1β and IL-6 detected by qRT-PCR, normalized to GAPDH. Data are presented as mean ± SD (n = 3). Statistical analysis was performed using one-way ANOVA followed by Tukey’s post-hoc multiple comparison test. **p*< 0.05, ***p* < 0.01, *****p* < 0.0001.
